# Neuroblastoma Presenting With Preseptal Cellulitis

**DOI:** 10.7759/cureus.47403

**Published:** 2023-10-20

**Authors:** Yu Furui, Shoji Saito, Kazutoshi Komori, Eriko Uchida, Takashi Kurata, Ei Shimazaki, Kazuo Sakashita

**Affiliations:** 1 Pediatrics, Shinshu Ueda Medical Center, Ueda, JPN; 2 Hematology and Oncology, Nagano Children's Hospital, Azumino, JPN; 3 Pediatrics, Shinshu University School of Medicine, Matsumoto, JPN

**Keywords:** pediatric infection, neuroblastoma metastases, nse, periorbital abscess, periorbital cellulitis

## Abstract

Neuroblastoma, a prevalent extracranial solid tumor commonly afflicting pediatric patients, exhibits a diverse spectrum of clinical presentations. Preseptal cellulitis, a childhood infectious ailment, typically demonstrates a favorable response to conservative antibiotic therapy. In this report, we present the case of a two-year-old female child with refractory preseptal cellulitis, ultimately leading to an unforeseen diagnosis of neuroblastoma. Early radiological assessment upon the onset of preseptal cellulitis serves the dual purpose of excluding severe complications and uncovering latent, rare pathologies when the initial antibiotic regimen proves ineffective.

## Introduction

Neuroblastoma stands as the prevailing extracranial solid neoplasm in pediatric patients, showcasing a wide spectrum of clinical presentations contingent upon the disease's status and the localization of the tumorous growth. Notably, approximately 10% of neuroblastomas commence with orbital lesions [1], characteristically culminating in subcutaneous hemorrhaging, colloquially known as "raccoon eyes" [2,3], and proptosis [1]. While preseptal cellulitis represents a relatively prevalent childhood infectious ailment amenable to conservative management, the timely identification of potentially grave complications such as post-septal disease, abscess formation, and cavernous sinus thrombosis is imperative to avert severe sequelae or fatal outcomes. In this context, we present a clinical vignette involving a two-year-old female with preseptal cellulitis who exhibited resistance to conventional antibiotic therapy, ultimately leading to the discernment of an underlying neuroblastoma diagnosis.

## Case presentation

**Figure 1:**
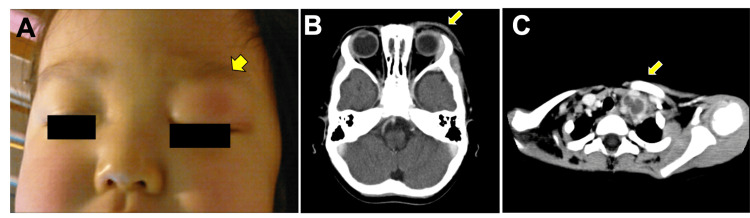
Clinical manifestations of the patient (A) Left supraorbital swelling in the patient. Arrowhead indicates the left supraorbital swelling and redness on admission. (B–C) Radiological findings of the patient. Enhanced computed tomography (CT) of the head and neck identified supraorbital skin thickening with irregular subcutaneous fatty tissue concentration (B) and an enlarged left supraclavicular lymph node (C).

A two-year-old girl presented at our hospital with painful and tender swelling in the left supraorbital lesion (Figure 1A) and a 39°C fever.

The patient had no history of trauma, upper respiratory infection, or dental infection. The left upper eyelid and supraorbital lesion were red and swollen. The eyeballs were normally placed, and extraocular eye movements were intact. Blood tests showed a neutrophil count of 7.6 × 103/µL, an elevated serum C-reactive protein (CRP) level (11.7 mg/dL), and a lactate dehydrogenase (LDH) level (641 IU/L). Blood culture testing was performed twice after admission but produced negative results both times. The patient was clinically diagnosed with preseptal cellulitis and was intravenously administered cefotaxime. The fever persisted, and left supraorbital swelling remained for 48 hours after treatment initiation. Enhanced computed tomography (CT) of the head and neck revealed supraorbital skin thickening with irregular subcutaneous fatty tissue concentrations without orbital cellulitis (Figure 1B). There were no signs of abscess, sinusitis, trauma, or dental infection. However, unexpectedly, the CT also revealed a mass lesion in the left anterior cranial fossa and an enlargement of the left supraclavicular lymph node with a low central concentration (Figure 1C).

**Figure 2:**
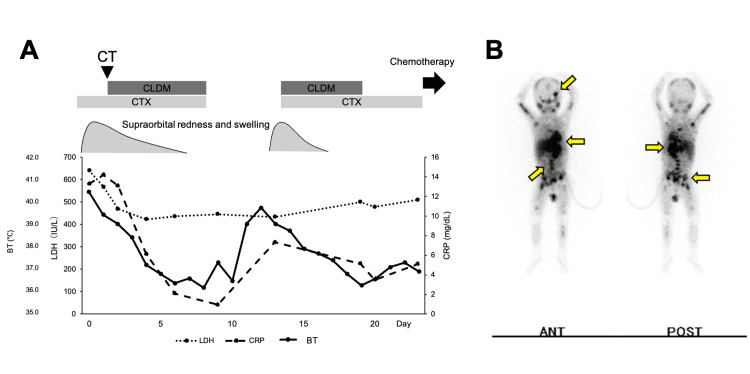
Clinical course presentation of the patient and disease dissemination in the patient by 123I-MIBG (A) The patient experienced the recurrence of preseptal cellulitis 11 days after the discontinuation of antibiotics. No antibiotics were administered at the time of chemotherapy initiation. (B) 123I-MIBG scintigraphy of the patient. The arrows indicate the origin of neuroblastoma in the left adrenal gland, left orbital metastasis, and bone marrow infiltration. CLDM: clindamycin; CTX: cefotaxime; BT: body temperature; LDH: lactose dehydrogenase; CRP: c-reactive protein; ANT: anterior view; POST: posterior view; 123I-MIBG: 123I-metaiodobenzylguanidine.

After the initiation of clindamycin, the patient became afebrile, CRP levels improved, and left supraorbital swelling was reduced. However, the serum LDH levels remained high despite the disappearance of the left supraorbital swelling (Figure 2A).

A re-evaluated blood test revealed a marked increase in the serum neuron-specific enolase levels (202 ng/mL). Enhanced chest and abdominal CT revealed an irregularly shaped mass in the retroperitoneum involving the left adrenal gland, revealing multiple calcified metastatic lymph nodes. A biopsy of the left adrenal tumor was performed, and histopathologic evaluation revealed a dense proliferation of small round cells, forming a Homer-Wrigh rosette, with some scattered mitotic figures and apoptosis. Based on the pathological findings, the patient was diagnosed with International Neuroblastoma Staging System stage IV neuroblastoma of the left adrenal gland. An abnormal 123I-metaiodobenzylguanidine (MIBG) uptake was also observed in the left supraorbital lesion (Figure 2B), indicating metastases of the neuroblastoma. The patient had no symptoms of hypertension, secretory diarrhea, bone pain, anemia, or opsoclonus myoclonus syndrome, which are known symptoms of neuroblastoma but only symptoms of preseptal cellulitis.

As the left supraorbital redness and swelling were resolved entirely, antibiotics were discontinued on day nine. However, fever and left supraorbital redness and swelling recurred by days 11 and 14, respectively, with an elevated serum CRP level. Cefotaxime and clindamycin were re-administered on day 14, making the patient afebrile and improving the left supraorbital lesion (Figure 2A). Finally, all antibiotics were discontinued on day 23, and chemotherapy was initiated for neuroblastoma. No recurrence of preseptal cellulitis was observed thereafter, even during the neutropenic periods of intensive chemotherapy. Multimodality therapy included intensive chemotherapy, tandem autologous stem cell transplantation, surgery, and proton beam therapy. The patient achieved complete remission following tumor resection. The patient stayed healthy in sustained remission for two years and 11 months after the end of therapy.

## Discussion

Herein, we present the case of a patient with metastatic neuroblastoma who was initially diagnosed with preseptal cellulitis. Our patient exhibited increased MIBG uptake in the left supraorbital lesion, indicating the presence of neuroblastoma metastasis in this lesion. Meanwhile, the patient repeatedly responded to antimicrobial treatment by resolving fever and reducing supraorbital symptoms, indicating the presence of bacterial infection in this lesion. Although we were unable to determine the precise pathophysiology owing to a lack of microbiological results, our findings suggested that neuroblastoma invasion of the periorbital lesion may be associated with the development and recurrence of preseptal cellulitis.

Neuroblastoma, a prevalent extracranial solid tumor in children, exhibits diverse clinical presentations contingent on disease status and tumor localization. Although approximately 10% of neuroblastomas begin with orbital lesions [1], the relationship between preseptal cellulitis and neuroblastoma has not been explored. To the best of our knowledge, this study is the first to report a case of neuroblastoma presented with preseptal cellulitis. As neuroblastoma is known to manifest various symptoms, clinicians should consider it a differential diagnosis when the clinical course is atypical during the treatment of common diseases.

Periorbital cellulitis is a relatively common infection of the eyelids and periorbital soft tissues in children, causing unilateral ocular pain, eyelid swelling, and erythema [1,4]. Although most cases can be managed with conservative antibiotic treatment as pre-septal cellulitis, 12%-18% of the cases of periorbital cellulitis reportedly required surgical intervention [4,5] for potentially serious complications, such as post-septal disease, abscess formation, and cavernous sinus thrombosis, which should promptly be excluded by radiological examinations. A recent management guideline proposed by Murphy et al. [4]. recommends performing CT when patients show manifestations such as central nervous system symptoms, gross proptosis, ophthalmoplegia, or no improvement 48 hours after treatment initiation, which may also help in the identification of clues for the diagnosis of rare diseases.

## Conclusions

Conclusively, we presented a case of preseptal cellulitis that was eventually linked to metastatic neuroblastoma. An early radiological examination should be considered if patients with preseptal cellulitis do not respond well to initial antibiotics.
